# Recent developments have made female permanent contraception an increasingly attractive option, and pregnant women in particular ought to be counselled about it

**DOI:** 10.1186/s40834-016-0034-1

**Published:** 2016-12-12

**Authors:** Douwe A. A. Verkuyl

**Affiliations:** 1 Leinweberlaan 16, 3971 KZ Driebergen, The Netherlands; 2 CASAklinieken, Leiden, The Netherlands

**Keywords:** Reproductive intentions, Unintended pregnancies, (Complications of) caesarean section, Ethics of female permanent contraception, Sterilisation regret, Climate, Religion, Less-resourced circumstances, Cultural diversification, HIV/AIDS, Contraceptive counselling

## Abstract

**Background:**

Despite the increased prevalence of reversible contraception, global unintended pregnancy rates are stable. Mistakes, method failures, side effects, alcohol, stock-outs, fears, costs, delays, myths, religious interference, doctors with other priorities, traditions and lack of health professionals may all factor in. Yet these unintended pregnancies ― nearly a hundred million annually ― cause much individual suffering, and in the long run, can aggravate conflicts, poverty, forced emigration and climate change. Presently, non-poor women postpone childbearing because of longer educational trajectories and careers. Sterilisations are therefore less often regretted or coerced. For poor-resourced women with a completed family, an unwanted pregnancy often has serious consequences, including crossing the (extreme) poverty line in the wrong direction, choosing an unsafe abortion, or even death. Caesarean sections (CSs), which currently stand at around 23 million annually, are increasing. On an “intention-never-to-become-pregnant-again” analysis, choosing a partial, and even more so a total bilateral tubectomy to be implemented during an ― anyway performed ― CS is by far the most reliable and safe contraceptive choice compared to meaning to start female or male sterilisation or any other contraceptive method later, and it reduces the chance of a future ovarian carcinoma substantially. CSs make subsequent pregnancies more dangerous. Simultaneously, they provide convenient, potentially cost-free opportunities for voluntary permanent contraception (PC): particularly important if there is no guaranteed future access to reliable contraception, safe abortion and well-supervised labour.

**Partial solution:**

Millions of women are within reach of attaining freedom from the “tyranny of excessive fertility” when they have a CS. Therefore, any woman who might conceivably be of the firm opinion that her family will be (over) completed after delivery should antenatally have “what if you have a CS” counselling to assess whether she would like a tubectomy/ligation. Yet many are not provided with this option: leading to frequent regret, more often than having been giving that choice would.

**Conclusion:**

Withholding antenatal counselling about the option of PC for in case the delivery might become a CS is very prevalent, yet often more medically risky, and morally questionable than when, even in labour, a doctor sometimes decides in the absence of earlier counselling, considering numerous factors, to provide the choice to undergo a concurrent sterilisation if s/he is convinced that would be in the patient’s best interest.

## Background

It seems appropriate to have a fresh look at female permanent contraception (FPC): the most prevalent (18.9%) contraceptive method globally; but on average little-used in Africa, the Middle East and Europe [1]. To reduce the global number of unintended pregnancies and associated misery, a larger role for permanent contraception (PC) would help. This will require a more proactive attitude from health professionals, and organisational changes.

## Reproductive realities have changed in favour of permanent contraception

Women in well-resourced settings, and often the prosperous and more educated in developing countries, are older compared to the previous generation when they (and their partner) decide that their family is complete [2, 3]. Increased age reduces the chance of regretted tubal occlusion (TO) and method failure considerably [4–8]. The proportion of mothers that accepts fatalistically that there might be a few more pregnancies/children after, say, age 36 ― which would also frequently interfere with their career ― seems to have shrunk. It appears that following the start of a new partnership (often with a man with children) after divorce or widowhood few older women do intend to have an extra set of children.

Child mortality, not seldom the cause of regretted PC, is at a historic low in many areas [9]. In fact, the increased use of reliable contraception for postponing, spacing and limiting is in itself the most important single factor reducing the mortality of mothers and their children [10–15]. If child mortality is high, it is risky to start PC the moment the family is seen as complete, although that is often the most, or only, workable opportunity [16–18]. It could later, however, leave one with what is felt as too few children or with no son or no daughter. When child mortality is rare, families would be more prepared to take that risk, resulting in much fewer unintended pregnancies/deliveries/maternal deaths. This releases family means for nutrition, attention, health care and education, as well as for extra resources per child on a national level, part of the demographic dividend [15]. If technically possible, perhaps some of the extra national reserves should be used for free of charge IVF after loss of a child following TO: this probably would strengthen the above virtuous circle. Even donors might become interested (one mobile IVF team per country even per region for this indication might be enough) if this approach would really result in far fewer unintended pregnancies. Another reason for the existence of large families is the need for old age care the demographic dividend might make it possible to organise a form of basic state pension.

TO never needs to be abandoned, unlike other methods, e.g., for cardiovascular problems including high blood pressure, breast cancer, heavy smoking, migraine, supply interruptions, inability to pay and pharmaceutical interactions, see Tables 1 and 2.

**Table 1:** Advantages of tubal occlusions performed during caesarean sections compared to hysteroscopic tubal occlusions performed later

1.	A TO during a CS is immediately effective.
2.	Patients can’t make post TO contraceptive mistakes.
3.	No need to check months later (ultrasound or X-ray) whether the TO was successful.
4.	When sutures are used during a CS (clips are irrational) the TO can be cost-free.
5.	One can be absolutely certain that the patient is not already pregnant.
6.	If the tubes are removed entirely ― easy during a CS ―, then method failure, including extra-uterine pregnancy, is extremely rare.
7.	If the tubes are removed entirely ― easy during a CS ―, then the future ovarian cancer incidence is likely to decrease by about a third.
8.	There exist no medical contraindications for a TO performed during CS.
9.	Technically, the procedure is virtually always successful.
10.	For women who turn out to deliver by CS and are certain that they want no more pregnancies, planning a TO during that CS will have a much lower failure rate than planning to postpone the TO (or partner’s vasectomy) until some months after delivery.
11.	If the tubes are just ligated, not removed, reconstructive surgery is possible.
12.	After postpartum discharge, the woman/couple very likely never needs to worry about contraception.

*TO* Tubal occlusions, *CS* Caesarean sections

Planning a mini-laparotomy for soon after a vaginal delivery shares, *mutatis mutandis*, with a CS/TO, ― when compared to an hysteroscopic TO later ― the advantages No. 1,2,3,5,6,7,9,11,12 and to some extent ― some postpartum bravery is needed, or theatre or staff might be not available ― No. 10. Compared to a laparoscopic TO later the advantages No. 5,9,12 and to some extent 6,7 and 10 apply to a postpartum mini-laparotomy

Compared to a hysteroscopic TO, an interval minilap TO (clips are with that approach also irrational) has, *mutatis mutandis*, advantages No. 1,2,3,6,7 and 11. In practice, many (perhaps 50%) hysteroscopic TOs seem to be performed under anaesthesia and not in an office setting, and they are in the US very expensive even more expensive than laparoscopic TOs [39]

**Table 2:** Advantages of tubal occlusions performed during caesarean sections over interval reversible contraceptive methods

1.	Immediately very effective, only a copper IUD has that advantage, and abstinence, a condom/diaphragm/coitus interruptus work also immediately, but not very effectively.
2.	Patients can’t make mistakes after starting the method.
3.	Technically, the procedure is virtually always successful. While, for example, IUDs are sometimes misplaced or fear/panic/pain stops the insertion procedure.
4.	TO is never abandoned because of side effects, stock-outs or rumours.
5.	The method never needs to be abandoned because the patient develops a contra-indication (e.g., high blood pressure, thrombosis, breast cancer, latex allergy, migraine, cirrhosis, cholestasis, smoking, pelvic TB/actinomycosis, fibroids or forgetfulness.
6.	One can be absolutely certain that the patient is not already pregnant.
7.	If the tubes are removed entirely ― easy during a CS, ― then method failure, including extra-uterine pregnancy, is extremely rare.
8.	If the tubes are removed entirely ― easy during a CS ―, then the future ovarian cancer incidence is likely to decrease by about a third, that is probably a larger reduction than resulting from the use of combined oral contraception.
9.	There exist no medical contraindications for implementation a TO during a CS.
10.	When sutures are used during a CS (clips are irrational) the TO can be cost-free.
11.	No further action is needed for method continuation as opposed to acquiring new pills, condoms or injections, replacing and removing IUDs or implants.
12.	Patients are independent of supply networks, i.e., there is contraceptive security. This also means that there are no more contraceptive costs.
13.	For women who are antenatally certain that they don’t want to become pregnant again, peripartum TOs will be followed by much fewer unintended pregnancies than will the patients’ intent to start a reversible method later.
14.	The partner can’t sabotage the method (throw away the pills, not cooperate with “natural” contraception or condom use) and he does not need to know.
15.	After postpartum discharge, the woman/couple likely never needs to worry (again) about contraception.
16.	Staunch Catholics will need to confess a TO as a contraceptive sin only once as opposed to the use of condoms, pills, rings or injections. Women can’t be made to stop TO. Some priests demand removal of an implant or IUD on pain of sacrament refusal, but circumventing a tubectomy with IVF is also a Catholic “sin” so priests can’t demand that.

*TO* Tubal occlusions, *CS* Caesarean sections

*Mutatis mutandis*, hysteroscopic TOs share with TO during CS six of the above advantages (i.e., No. 4,5,11,12,14 and 16) vis-à-vis reversible contraception

*Mutatis mutandis*, laparoscopic TOs share with TO during CS ten of the above advantages (i.e., No. 1,2,4,5,7 ― but not that easy, 8 ― but not that easy, 11,12,14 and 16) vis-à-vis reversible contraception

There is convincing evidence that removing part of the tubes ― most likely the more the better ― significantly reduces the incidence of ovarian cancer, one of the deadliest malignancies [19–22].

More and more women have careers outside their home, encouraging a decision on when they want their childbearing years to end dependably, and most don’t consider a termination of pregnancy (TOP) ― about 56 million annually and increasing in absolute terms, of which about half are unsafe, and 73% are obtained by married women [23] ― a triviality.

Presently, more women are self-confident and educated, and therefore less likely, it seems, to be coerced into having a TO.

## Caesarean sections can be excellent occasions to provide permanent contraception

In most parts of the world, caesarean section (CS) rates are growing [24–26]. CS rates are higher if women are older, have been treated for infertility and have higher BMIs [27–30]. A CS, if it completes the family, provides a very convenient, cost-effective and the safest ― possibly even including vasectomy ― PC opportunity. Conversely, a laparoscopic and also a “minilap” interval TO can become somewhat more dangerous after a previous CS because of adhesions [31, 32]. But more important these interval procedures including hysteroscopic TOs are in practise difficult to organise and therefore result in much higher failure rates if analysed on an intention to have PC basis [33, 34]. Similarly, non-implemented firm intentions to have an “interval” vasectomy occur following perinatal pledges by partners.

About a quarter of women in the European Union (EU) have their last child via a CS [25], while in the United States (US) in 2013, 43.1% of women aged ≥35 years who delivered had a CS [2]. In 2012 an estimated 22.9 million CSs were performed worldwide, around 19.4% if the 118 million live births are used as denominator [26]. If 10 million of those CSs would involve women who would like to stop having children and 50% of those would like and receive a TO and these women were on average 32 years old, then, 5 million times 18 years is 90 million couple years of protection could be generated per year, each year. This is twice what is created by the approximately 160 million 3 monthly contraceptive injections given annually and only a little less than what is suggested by the 1.3 billion pill-strips supplied yearly [1], ― while there would be far fewer failures, (hidden) costs and side effects. These CS/TO projections are not completely unrealistic, it is about what happens in Brazil and to a lesser extent ― perhaps explained by the higher vasectomy rate there ― even the US.

Scar-related pregnancy complications are on the rise, so much so that regional centres of excellence are established to cope with them in the US for example [35–38], therefore the prevention of unintended post-CS gestations is especially important, even more so under non–high-tech circumstances. Induced abortions also can become more dangerous (placenta praevia or increta, weak scar, caesarean scar ectopic pregnancy) when the uterus is scarred.

TOs implemented during a CS or just after a vaginal delivery (8–9% of all deliveries in the US are combined with a TO: involving around 15% of all deliveries of second and higher children) have probably the lowest FPC failure rates, the largest prospective study shows: 7.5/1000 procedures cumulatively over 10 years [7, 8]. Following ― non-electrocautery ― interval laparoscopic TOs this rate was shown to be 36.5/1000 [8]. There seems to be not much difference in typical pregnancy rates after a laparoscopic clip application and a hysteroscopic TO [39, 40]. Interval tubal cautery is very effective but increases the ectopic pregnancy rate in the rare cases the method fails [8]. Following a peripartum bilateral total salpingectomy (BTS) the contraceptive efficacy is very likely to approach 100% [7].

As mentioned, TOs with clips, rings, cautery or sutures ― just like having one or more children, lactation and the use of combined hormonal contraceptives ― reduces the subsequent ovarian cancer risk [7, 19]. Probably, a BTS, the fimbriae included, reduces the risk even more [7]. The ovarian cancer protection effect of TO is not, unlike that of hormonal contraception, associated with some increase in the breast [41, 42] and cervix cancer risks nor the suspicion of an increased HIV acquisition/transmission risk (vide infra), nor, conversely, with a decrease in colon and endometrial malignancies. Until there are further ― preferably randomised ― studies (however unlikely these are to be performed), the American College of Obstetricians and Gynecologists (ACOG) does not for the moment support changing the TO methodology: i.e. surgeons are advised to continue to observe and practice minimally invasive techniques for FPC [43, 44]. Even so, BTSs during CSs and soon after vaginal delivery would not need a larger incision, would have not more complications and would prolong the operation, on average, 10 min [7, 43, 44]. This probably applies also to interval mini-laparotomies as performed (often under local analgesia) in less-resourced settings where ovarian cancer has an extra serious prognosis. The issue is still debated but it is at the stage that, when feasible, patients wanting FPC should be counselled about the choice between a classic, a hysteroscopic TO and a BTS or, even better perhaps, offered inclusion in a randomised trial [44]. Noteworthy, in routine practice premenopausal hysterectomies for benign indications are increasingly combined with BTSs for ovarian cancer prevention. For the foreseeable future, performing opportunistic BTSs seems the most promising approach for reducing the ovarian cancer mortality meaningfully, much better than screening and treating, and certainly more cost-effective [45].

While in North America the TOP rates for unmarried women are, if anything, higher than in Europe, for married European women these are 2.7 times the North American rates [23]. This is very likely related to PC because PC (male plus female) is 6.4 times more prevalent in North America (36.0%) than in Europe (5.6%) [1].

Finally, there are many areas where the (health) resources are so limited and/or the costs of interval TO so high that FPC can only be provided in practice for the average woman, during a CS and maybe post-partum or post-abortum [14, 16–18, 33].

## Doctors ought to be stewards of finite healthcare resources

Among other factors, political developments, new therapeutic options, aging or growing populations, enhanced public expectations, HIV, Zika and shrewd, not seldom unethical, marketing strategies could make health care even more expensive, which might in turn affect the resources available for basic health care and other areas of public spending [14, 46, 47]. Therefore, providing PC in combination with other procedures if the opportunity presents itself makes financial and ethical sense as long as there are few regrets [48]. Moreover, if the costs of the typical failure and discontinuation rates of other methods and the need for further medical attention are taken into consideration, then, whether combined with another operation or not, FPC and vasectomies (unacceptable in some cultures, fewer method failures, but not immediately effective and pregnancies from irregular partners occur) are often anyway the most economic contraceptive options excluding perhaps, depending on the circumstances, IUDs [10, 49, 50].

## The smaller the desired families the more difficult it is without PC to prevent unintended pregnancies

With the current global total desired fertility rate of little above 2 children per woman ― although governments sometimes interfere [51] ―, most women (couples) will spend 25–30 years trying not to become pregnant and have on average around 0.8–1.0 TOPs [23, 52].

The development of a convenient, reversible, non-client dependent, reliable contraceptive method with few, if any, negative side effects is therefore a priority, Nobel-Prize Worthy, and the-least-we-should-do, now that landing a probe on a small lumpy comet, after travelling 6.4 billion kilometres through the Solar System, is achievable and Higgs Boson has been sensed. Presently, annually, an estimated 33 million ― and increasing ― women worldwide experience an accidental pregnancy while using contraception [23, 53]; more women than would have been unhappy if the Boson had not been found.

It seems likely that future generations will succeed in making the inability to become pregnant the default position (for men and/or women), i.e., starting a pregnancy would require some technical, administrative and perhaps spiritual and ceremonial efforts. Though still flawed in important ways ― it is still of little use to women who want to postpone, space or who are undecided ―, an easily accessible PC method after the family is completed, with free IVF/reconstructive tubal surgery/ICSI or artificial insemination with stored semen for those that develop serious regret, appears in theory a cost-effective, rational step in the right direction.

## There are problems with the reversible methods

Implants and IUDs have large discontinuation rates, so have pills, rings, patches and injections [54–58].

Even in the US in 2011, a country where PC is quite prevalent (female 23.6%, male 12.7%) [1], or, with a different denominator, 47.3% of married couples (TO 30.2%; vasectomy 17.1%) [4], nearly half (2.8 million) of the around 6.1 million pregnancies was unwanted or mistimed and of those 54% is attributable to non-use, 41% to inconsistent or imperfect use, and 5% to contraceptive failure [57]: reversible client-dependent methods are of course very disproportionally involved [49]. Of the 4.0 million US births 1.5 million (40%) were at conception unintended of which 720,000 (18% of all births) were not merely mistimed but the mothers involved had not wanted the index pregnancy nor a pregnancy anytime in the future [57]. Poor women had an unplanned birth rate nearly seven times that of higher-income women [57]: A tiered system in which women with money have options and those who haven’t, more babies. This works naturally also the other way round: unplanned pregnancies often make women/families poorer.

The above is very unfortunate, but it would cause hopefully pandemonium if the resulting TOPs, around 1.1 million in 2011 [58], would be as unsafe ― with a case fatality rate of 1:192 ― as in sub-Saharan Africa (SSA) [53]. In the US 5700 women would die annually, and many more would have lifelong disabilities. However, with the new US president, with Republican majorities in the House, the Senate, and in two-thirds of the state governors’ mansions and uncertainty about the future composition of the Supreme Court a move to end Roe v. Wade seems a possibility. Of course, many of the unintended pregnancies in the US involve women whose family is not completed but it demonstrates how difficult it is to avoid unintended pregnancies without PC. In fact, in countries where the proportion of women using a contraceptive method very clearly outweighs the proportion with unmet need ― hopefully the future everywhere ―, often the greatest numbers of unintended pregnancies come about as a result of incorrect or inconsistent use [14]. Long-acting reversible contraceptives (LARC) perform better but need well-trained often expensive health professionals, often in poor-resourced countries in short supply.

While dispensing oestrogen-progestin combinations requires from health staff the least effort, time, motivation and training these methods have, apart from high typical failure and discontinuation rates [14], not seldom non-negligible side effects in the last 15 years of a woman’s fertile lifespan [31, 59, 60]. Even the safest combination is associated with 2.5 times the thromboembolism rate seen in non-pregnant non-users of similar age and health, while age (and of course smoking) has a large impact on the *a priori* rate [31, 59]. This means that the risk-benefit analysis for these combinations for a specific woman will nearly always show that their use is less dangerous than a pregnancy, but the risks compared to using PC, increase with age. In some countries with low PC rates many women above 40 years still take the pill [60, 61], while in others there is often no functioning sphygmomanometer in the clinics that supply contraceptive tablets and/or injections.

Although 10–20% of users will discontinue LNG-IUS because of side effects, the method, if affordable, is often popular with completed families ― also because of the positive side effects. For an undefined percentage of Muslim/Orthodox Jewish/Animists families, LNG-IUSs (and implants) are unacceptable because of the associated cycle disruptions [62]. The non-evidence-based opinion that the dominant mechanism of action of all, or only the copper, IUDs is inducing abortions precludes their use for a sizeable portion of monotheists.

Fewer women will develop endometrium cancer with LNG-IUS use but, conversely, there might be a slight temporary increase in breast cancer incidence of a similar magnitude as seen with oral contraception [63, 64]. If confirmed this will be one of the “developments” mentioned in the title of this contribution.

## Increasing cultural diversification

There is increasing diversity in many countries. Women who hail culturally from, for example, Latin America, The Caribbean, Suriname, India, Thailand, Iran, Cape Verde, South-Africa, Turkey, North America or China, where PC for a completed family is well-established [1], might be reluctant even anxious to use (some of) the unfamiliar, i.e., never used by their mothers and peers, reversible methods entrenched and popular elsewhere, costs are also a factor [10, 14, 50, 56, 57, 65–69]. Moreover, the continuous motivation and discipline needed for injections, condoms and pills are a problem for many [14, 70, 71]. Alas it is very easy for women to find support for their anxieties on the internet. Doctors unfamiliar with providing specific methods like PC, IUDs or implants often hide their lack of expertise by rubbishing the method and tarnishing its reputation, sometimes for years. Obstetricians and midwives might take it mistakenly for granted that immigrants or refugees who would like a peripartum PC will in time take the initiative. In many cultures vasectomies are unacceptable or just rarely performed (Eastern Europe 0.2%, Africa and Middle East both 0.0% prevalence) [1], leaving women with contra-indications, side effects or “afraid of hormones” [67] and with objections to devices in their wombs [62], and without realistic access to interval FPC, no good alternative. The above partly (confounded by income, education and ethical/religious outlook) explains the very large variations detected in well-resourced countries in TOP rates related to poverty and cultural backgrounds [57, 65, 72–74]. These differences escalate with repeat abortions [72–74]. At the same time immigrant women in rich countries can have high, especially emergency, CS rates [75]. Discussing the FPC option in time during the antenatal period seems therefore mandatory, but happens seldom in some countries [5, 48, 76]. High abortion rates of immigrant women can also be misleading. They suggest that families are not larger than desired. However, young women often give “religious reasons”, as grounds for TOPs, i.e., pregnancies (or rather sexual relations) before marriage are not allowed. But older multipara, also those with medical conditions warranting at least thorough preconception care, mention “religious imperatives” often as reason for continuing an unintended and unwanted pregnancy.

## Without family planning one needs migration planning

It has become apparent that fossil energy consumption needs to be limited, and that land and water for food and fuel production are finite and climate change will ― and probably is already ― affect food security [11, 77–79]. Therefore, sustainable, equitable global development ― including a catch-up operation for those left far behind ― seems even more unrealistic if unintended pregnancies ― 85 million annually, of which around 42 million (other studies find different numbers, e.g. 56·3 million [23]) become TOPs, and 32 million (more than a quarter of all live-births) births [12, 53, 80] ― are not more successfully avoided. Most new-borns “just replace” the deceased, but the 32 million play a large part in population growth, including of course those that are born earlier than intended (mistimed). Alas, prominent climate activist Naomi Klein in her important book “This Changes Everything” [81] suggests a focus on population is a “distraction” in the fight against climate change. For many, mostly the rich, it is indeed a pretext not to change their fuel burning lifestyle. However, the problems related to fast growing populations (migration, war, poverty, unemployment, famine, national and international political clashes and failed states) lately also seriously distract governments and make it more difficult to unite for climate change mitigation. Immigration side effects affected the elections in the US and very likely tipped the balance direction Brexit: huge distractions. The immigration/refugees upheavals are much less abstract for many voters than climate change. Klein is also dismissing, against evidence to the contrary, the estimated 225 million women in the developing world, and those not well quantified in well-resourced circumstances, who want to avoid pregnancy and are not using a modern method of contraception [1, 3–11, 80, 82], as a factor that needs urgent addressing. For example, a study from Oregon University claims that, in well-resourced circumstances, the lifelong climate-stabilizing efforts (vegetarianism, use of local products, (re)cycling, good isolation, electric cars, solarpanels, windmills, no air miles, etc.) of twenty people are cancelled out by the birth of one extra child [83]. It follows that in the US the dedicated lifetime climate-conserving efforts of 14 million people are neutralised as the result of the above-mentioned 720,000 unintended, not merely mistimed, births resulting from contraceptive failure and non-use every year, [57, 84–87]. A study commissioned by the UK-based charity Population Matters together with Lancaster University asserts: “Reducing future energy demand by preventing unwanted births and hence lifetimes in developed as well as developing countries is far cheaper — US $1.11 per ton reduction in CO2 emissions — than any renewable energy alternative. The benefits multiply in perpetuity via each never-existing person’s never-existing descendants” [88]. More people means more fossil fuel intensive fertiliser use, irrigation, heating, vicious circle air conditioning, and transport, and less room for biofuel production (probably needed for sea and air transport for a long time to come, even after cars are powered renewably electric) because it competes with food production, not to mention nature.

Klein’s position is possibly an attempt not to antagonise the Vatican, a powerful climate ally. It is estimated that the Catholic Church operates more than 5300 hospitals worldwide [89], representing both an enormous amount of alleviated suffering, and unnecessary misery on the individual and collective level by undermining modern contraception and sex education via all possible platforms [11, 90, 91]. A recent example, women in some regions in Latin America were told by health officials to postpone pregnancy because of Zika, and regional religious officials told them, not publicly contradicted by the Vatican, not to use modern contraception and have, regarding the poor, often the influence to make them. Especially poor families, unable to access affordable health care and paid sick leave, will suffer enormously raising a child with microcephaly, and there is of course the suffering of the child. Why does the ACOG or the Polish College not advise its members to boycott hospitals that refuse to allow perinatal sterilisations or the provision of other modern contraceptives for that matter?

An estimated 230 million births annually (1.7 times the number of livebirths) and 270,000 maternal deaths are already “sinfully” averted by current global contraceptive use [12], imagine the sin-generating misery if they weren’t. Another 21 million births (and at least 26 million TOPs) would be probably avoided if the unmet need for contraception was satisfied [80]: more if people had access to a more effective method mix, including PC [92]. In Latin America 56% of the pregnancies are unintended apparently the highest rate of all continents [53, 80]. This is no doubt intimately related to the desire for small families combined with the traditions and habits formed under a strong religious influence, e.g., non-evidence-based opinions about the serious dangers in this and the next life of using modern contraception. However, although still mostly illegal, TOPs (estimated 6.6 million [23] in Latin-America) are unlike in the past ― church is now ignored and misoprostol available ―, rather safe, about as safe as in Eastern Europe and in fact, significantly less dangerous than women bringing their pregnancies to term [53].

It seems also likely that the Vatican’s attitude to modern contraception is considered by other major religions as partly an attempt to consolidate or increase power by numbers. This stimulates in my opinion, it is very difficult to prove, an extra pronatalist attitude of non-Christian ― even protestant (certainly in the past) ― religious leaders. This would make the Vatican, for many outsiders representing Christendom, partly responsible for the climate-change and population-pressure-related misery. Besides, it is also not implausible that immigrants with high fertility rates will encourage higher birth rates of the locals in an attempt to preserve the dominant culture (“Leitkultur”).

There are large regions where, among other factors, the density and rapid increase of the population impede for many access to the basics of a decent life, and where climate change might push many more over the brink [93]. If degrading plots or grazing areas have to be shared by successive burgeoning generations (1 hectare/household, 25 years later ½ hectare/household, 25 years later ¼ hectare etc.) and there are few other income-generating opportunities, then grinding misery seems unavoidable if total fertility rates stay (much) higher than two. Even more so if child bearing is not postponed to later in life and therefore 3–4 generations have to live simultaneously from the same food source instead of 2–3. This is the case in much of Africa, non-oil rich Middle East, Afghanistan, Pakistan, and areas of India, Latin America and the Philippines. The 48 Least Developed Countries (LDCs) had an estimated population of 954 million in 2015, this is projected to double to 1.9 billion persons by mid-century and increase to 3.2 billion in 2100 [94], while the number of women with an unmet need for modern contraception is increasing in those very countries [50].

One should also mention that areas with high birth rates and, therefore almost by definition with many unemployed men of military, criminal gang and drug-cartel age, tend to be in turmoil. This is no doubt related to the current refugee tragedies [85, 93]. There are according to the UNHCR more than 65 million forcibly displaced (refugees, asylum seekers and the internally displaced) people ― the largest number since the Second World War and four times more than a decade ago. Besides, military actions burn much fuel and insurgencies interfere with, for example solar farms or conservation projects. Not only can rapid population growth and climate change related droughts [93] and coastal submersions [85] result in (civil) war, in addition loss of all hope for the future at home also creates refugees [78]. It seems, for example, elementary to try leaving Nigeria (and/or to have an offshore nest egg) if the population will indeed increase from 182 million in 2015 to, medium variant, 752 million in 2100 while oil income will probably evaporate even more and while there are already many very poor, malnourished and hungry Nigerians [94, 95]. The above population projections could easily be underestimates because use of modern contraception has not increased in the period 2008–2013 in Middle and West Africa while the above projections assume decreasing fertility rates [11, 49]. Conversely, the associated future increased mortality might cause overestimation. Increasing Mediterranean tragedies seem a given.

When state authority fails like in some sub-regions in Latin America, Afghanistan, Central African Republic, Mali, Burkina Faso, Yemen, Haiti, Iraq, Syria, South Sudan, Chad, North-East Nigeria, North-East Kenya, East Pakistan, Libya, Burundi, the Democratic Republic of Congo, Ivory Coast and Somalia, it is often too late, even downright dangerous, to (re)start a reproductive health programme [96] while warlords, factions and ministries of defence need cannon fodder (51), and also more radicalised religious interpretations might threaten contraception providers.

In, for the time being, still well-resourced circumstances, however, the feared decrease in productivity (and military capability) and the aging of the population as result of low birth rates and increasing life expectancy will possibly be resolved by robots ― if not by the (children of) refugees who might take some time to adjust to the (re) productivity in their new homes [65, 72]. Besides, although Japan is an extreme example of an aging population, and resists immigration and is very active in robot development, it seems that hundreds of millions would be quite happy with the Japanese standard of living.

Currently, of course, almost everywhere, (youth) unemployment is a much larger problem than lack of personnel. In any case an economic model with ever-increasing productivity and consumption needing, and at the same time driven by, a growing population, seems for the foreseeable future unsustainable [81]. Professionally, for doctors, midwives and nurses the low-hanging refugee/climate-change/malnutrition/misery/failed-state and war prevention fruits seems to be assisting patients optimally to prevent unintended pregnancies, i.e. this is probably a much more effective contribution than exchanging a SUV for a Prius. One could argue there is a moral imperative for the above fruit harvesting because “Medical science bears much responsibility ― albeit without intent ― for the population “explosion” of the past 200 years” [11]. Many a misery prevention opportunity, including providing the option of PC in case of CS or vaginal delivery, is missed [5, 14, 18, 90].

## TOs delayed are often TOs denied

Frequently, if women and their partners have used reversible contraceptives for 5–20 years or so, followed, often in quick succession, by two successful pregnancies, then, for many, but for the fact that first an operation is needed, being protected by a vasectomy or TO is a desirable state of affairs. A small study ― of course not randomised ― in the Netherlands showed that women depending on PC were the most satisfied contraceptors [97] and an Australian study found ― “should be included in the counselling” ― improved sexual lives of women having undergone TO [76].

Globally, there is however a shortage of 2.2 million surgeons, anaesthetists and obstetricians [98]. It is estimated that the global needs-based shortage of health-care workers will be more than 14 million in 2030. Billions do not have access to safe, affordable surgical and anaesthesia care when needed, let alone the provision of implants, IUDs, safe abortion, postpartum and interval PC, which is very often not perceived by health staff, mistakenly in my opinion, as an attention demanding emergency [92, 96, 98, 99]. Just within the EU there are vast inequalities in access to reproductive health goods and services [66]. The lack of well-trained contraception providers is particularly severe in regions with a high burden of unsafe abortion.

Even under better-than-average conditions it is not easy for a woman to realise an interval TO because of anxiety, costs, travel, lack of facilities and confidentiality, bureaucracy, time off work needed and loss of income. In comparison, it is rather easy to improve knowledge, attitudes, guidelines, software and practices related to opportunistic TOs. To pressure a woman to have a concurrent TO if she will undergo a CS anyway is nearly [100] always unacceptable [76, 96, 101, 102]. Yet denying women/couples that choice is routine in many areas, especially in continental Europe, many Islamic countries and in non-southern Africa [1, 5, 17, 18, 76, 92, 96, 101, 102]. This is also a form of coercion. Curiously, this compulsion doesn’t result in litigation, public protest or disciplinary boards. Test cases are urgently needed. For example, concerning a 40-year-old woman who completed her family via a CS without being provided with the TO option, and who later because of a scar-related placenta accreta had a life-threatening delivery, TOP or miscarriage, or a fatal pulmonary embolism on the pill. Moreover, reproductive coercion in the form of non-consensual sexual intercourse or sabotaged contraception, pregnancy pressure and control of reproduction by an authoritarian, patriarchal or abusive partner [103], or refusal of a Catholic hospital to allow TO with a CS, or political interference with access to contraception or safe abortion, is much more prevalent [51, 91] ― although not an excuse for health professionals to also flout ethical standards ― than coerced PC.

A TO has obviously one large disadvantage: it is difficult or impossible, depending on the circumstances, to have more children if a woman changes her mind. On the other hand, in the unlikely event that it would be possible in future to close the tubes ― or vasa deferentia for that matter ― via a reversible mechanical system without increasing failure rates, side effects and costs, then the ovarian cancer prevention advantage of TO would disappear.

It is initially frequently better to introduce FPC in a specific country, sub-culture, hospital or religious denomination if one starts with CS/TO. It is easier to accept, even for health staff, because there is often ― especially with repeat CSs ― a medical-indication aspect to it [35–38], and no extra resources are needed. Women can often tell their mothers-in-law, peers, husbands and confessors quite truthfully that the doctor thought it safer not to have more pregnancies. In Ireland, the Dutch Catholic (before that the protestant) hospitals, Brazil and southern Africa and probably many more places this stimulated the initial approval of FPC by ― even religious ― opinion leaders. That was followed by the acceptance of interval TO as one of the reasonable contraceptive options. Before that, quite some, mostly well-to-do, women had to assert in desperation, and the gynaecologist often knew they pretended, that they had, for example, debilitating periods, in order to qualify for a hysterectomy with as “side effect” PC. A more dangerous, operation of course, with more subsequent side effects. In Ireland, when non-“natural” family planning was still illegal, quite risky caesarean-hysterectomies were performed more frequently than seemed medically indicated to circumvent the Church’s dictates [104]. Perhaps the realisation that TO reduces the ovarian cancer risk will stimulate a reappraisal of FPC in some countries.

In well-resourced settings, hysteroscopic TOs ― to be implemented at least six weeks post-delivery ― are currently commonly put forward by obstetricians as the smart, better timed and considered, office-based, non-surgical option, and therefore more preferable than peripartum TOs, for those soon to have a completed family [5, 33, 48]. However, although a good solution for some, as mentioned their failure rate is much higher especially if analysed on an intention to treat basis [33, 34]. A TO method that requires optical instruments is anyway less suitable for introducing PC in a specific country, also because of the training, capital outlay and disposables needed ― to be recuperated via the patient one way or the other ― and maintenance of equipment required. Moreover, a few complications can ruin the acceptance of this method, or perhaps even TO in general, in a certain area for years. Hysteroscopic TOs have lately a somewhat negative professional and lay press [39, 40, 105] which will reduce the number of women actually turning up for PC a few months after a delivery even more.

Similarly, many US Medicaid beneficiaries (a very large proportion of women who deliver) miss a desired peripartum TO due to all sorts of cumbersome barriers/red tape/misplaced forms and are when Medicaid runs out, soon after delivery, unable to afford a TO or any other reliable method ― higher income women do not encounter this obstacle [106, 107]. The protocols/administrative hoops are meant to protect minorities, women with mental problems and the intellectually challenged. Very understandable in the light of what happened in the past with eugenic TOs. However, medical ethical standards have since evolved considerably in many jurisdictions and these rules, probably, do now more harm than good [106]. The fallout is estimated in the US alone to be up to 62,000 unfulfilled requests for postpartum sterilization, 10,000 abortions and 19,000 originally unintended births annually, at a public cost of $215 million [17, 106]. Another US analysis showed that nearly 50% of women who missed an intended peripartum TO experienced an unintended pregnancy within a year [107]. Correspondingly, denying a woman in Oregon, USA who asks for an abortion because of a completed family a concurrent TO, results on average, over the next five years, in 1.3 unintended pregnancies and an additional $4,152 in direct medical costs, a computer simulation indicated [108].

## Planning and counselling

In general, health professionals ― GPs, obstetricians, midwives, paediatricians, psychiatrists, geneticist, district doctors, neurologist, dermatologists, oncologist, infectious disease specialists, cardiologists, rheumatologists, urologists, health visitors, refugee support staff [109], surgeons [110], etc. ― need to assess the reproductive intentions of their patients. Perhaps they can help preserve gametes or prevent unintended pregnancies, pharmaceutical interactions with hormonal contraception, congenital abnormalities, unhappiness and/or the interruption or termination of education.

**Fig. 1:**
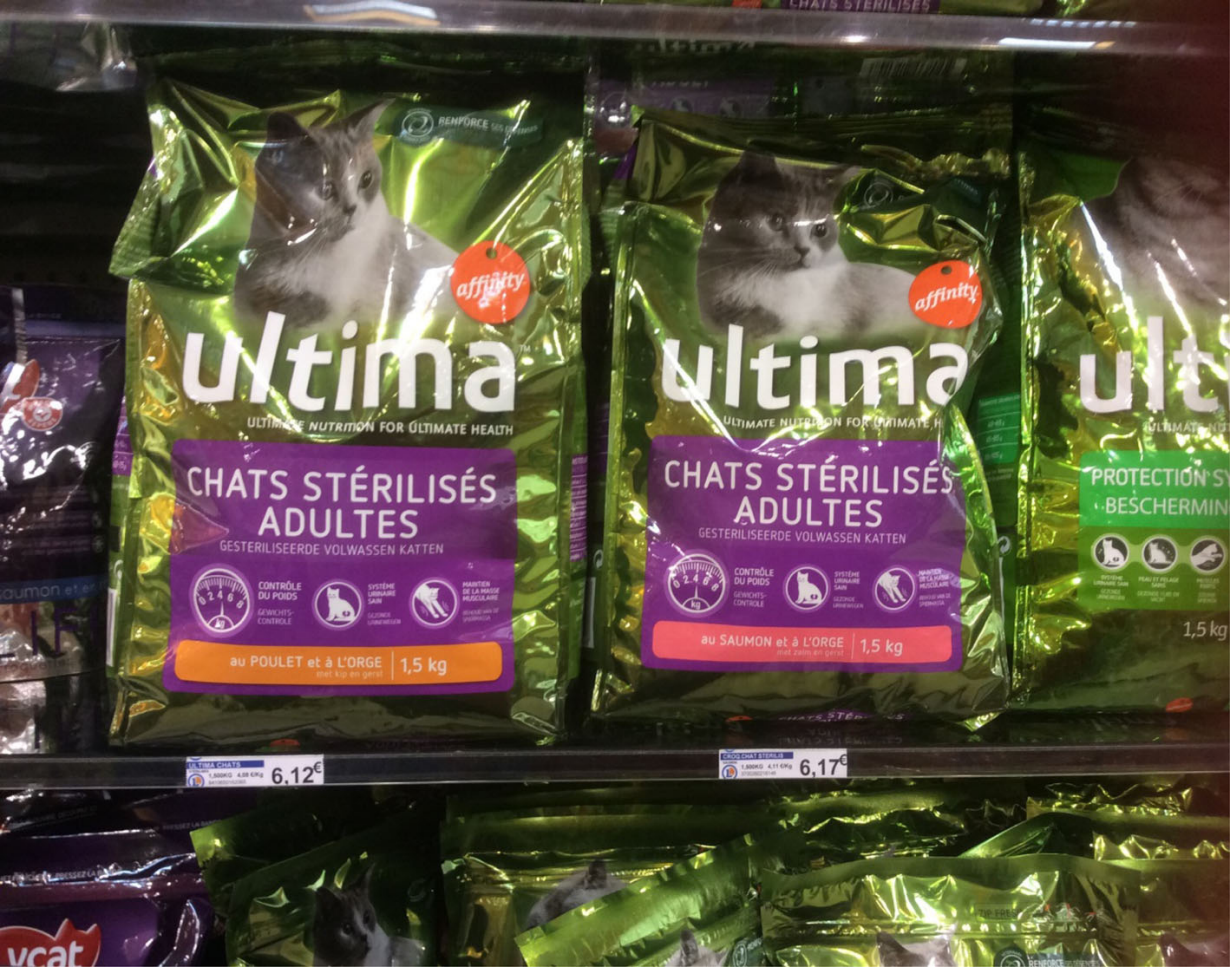
Dastardly subtle, misleading, anti-sterilisation publicity seen in every supermarket in Belgium and France. Apparently after “sterilisation” mammals need a special diet otherwise they become fat and indolent. Not exactly innocent when nearly half of the European adults have “inadequate” or “problematic” health literacy [170]

Providing the option of a TO, should a CS later turn out to be necessary, requires, if at all possible, some weeks before the due date, routine, gentle, delicate checking whether perhaps the patient/couple is of the firm opinion that, if all goes well in the near future, the family is thought to be complete. If that is indeed the case, proper further counselling is needed and reviewed not long before and even during the actual delivery in the light of the then-available information. Counselling includes in some countries with misinformation about this subject in every supermarket, talking about cat food and the difference between castration and TO [see, Fig. 1]. The TO counselling should be standard procedure in most third trimester pregnancies according to the International Federation of Gynecology and Obstetrics (FIGO)’s Committee for the Ethical Aspects of Human Reproduction and Women’s Health [96, 101]. The discussion should be subtle because some women/couples see this as an intrusion of their privacy and/or a sign of disapproval if they have more children than the local average, or as distrust of their parenting abilities, or, if there is a difference in background, even as a sign of racism, or religious or HIV discrimination. If the relevant mothers ― e.g. in the EU, 53% of the women seen in the antenatal clinics are expecting their ≥2 child [25] ― know that this checking is routine ― just like inquiring after smoking, breast feeding intentions or suggesting a HIV test ―, then few will feel insulted. There are also women who volunteer, following the slightest encouragement, especially when they expect their ≥3 child that the pregnancy was unintended and that they certainly don’t want even more children.

In circumstances where a hysteroscopic, laparoscopic or minilap TO some weeks-months after delivery is in theory a feasible option, obstetricians/midwives should be very clear antenatally when counselling prospective TO candidates: interval TOs have much higher failure rates than peripartum TOs, overwhelmingly so if analysed on an antenatal intention-to-have-PC-before-ovulation-returns basis, also because they are often much more expensive [18, 33, 34, 48]. In the Netherlands, a hysteroscopic TO costs at least €1450 including follow-up (the net monthly wage of a junior police officer/junior doctor, and the procedure (unlike IVF) is not covered by most (mandatory) insurance plans, just like the other reliable contraceptive methods are not. Ironically, TOPs are paid for by the state. In the US, the median of the total charges for a hysteroscopic TO is an unbelievable $7832, and for a laparoscopic TO $5068 [39]. One can imagine some patients getting rather disturbed if the obstetrician or hospital [106, 107] had denied them the CS/TO choice. The doctor could also have a financial conflict of interests.

Sometimes a TO counselling opportunity arises if a woman is to have an ovarian cystectomy, a cholecystectomy (gallstones classically bother fairish, fortyish, fertile females), removal of a perforated IUD, treatment of a miscarriage or extra-uterine pregnancy, or bariatric surgery ― the latter also a good idea because after, depending on the type, such surgery, absorption of oral contraceptives can be reduced, and unintended pregnancies because of unanticipated restored fertility and perhaps resumed dating, occur frequently and are not without risk [110].

In many, but not all [76, 111], studies, interval procedures have somewhat lower regret rates than perinatal TOs. However, most of these studies are somewhat dated and therefore they involve women on average younger and perhaps less self-confident therefore more coercible than in general would presently be the case. Moreover, only the very keenly motivated (sometimes prepared to pay €1450―$7832), tenacious and rather courageous will succeed, often against odds, in having an interval TO. This in itself could explain lower interval TO regret rates. Using this difference in TO regret rates as an argument to discourage CS/TO will of course dramatically increase the missed-a-TO regret rates. TO soon after a vaginal delivery still requires courage and often payment, but other barriers like transport, lack of baby sitters, difficult communication, exposure to strangers and unfamiliar surroundings are less in evidence and moreover BTS is easy and that will result in optimal ovarian cancer prevention. Besides, coercion is quite unlikely [96].

Sometimes, specifically in areas with few resources, the removal of a retained placenta after a vaginal delivery in a woman of high parity, if she has anaesthesia anyway, can be combined ― especially when antenatal assessment of her reproductive intentions revealed that her family is (more than) complete ― with a TO/BTS via a mini-laparotomy. Adherent placentae often recur in subsequent pregnancies and can result in catastrophic bleeding. Ectopic pregnancies are also seen in women of high parity with completed families, and there is a serious risk of recurrence.

How to balance the risk of unintended (sometimes dangerous) pregnancies later with the risk of regretted PC is in theory easiest addressed by giving the relevant women/couples the choice, after proper counselling [96, 106]. Yet, sometimes giving a clear recommendation based on a reasonable understanding of a woman’s own preferences and potential future risks is preferable, for which the term ― extra suitable in this case ― maternalism, as opposed to paternalism, has been coined. [112, 113]. A study in the Netherlands, where women on average have their first child when they are 29.4 years ― average EU 28.7, Spain, Italy and Switzerland >30 years [3] ― demonstrated that a policy of not initiating counselling about a potential TO in combination with a potential CS for the ≥2 child, was 62 to 186 times more likely to end in regret, than facilitating an informed choice did [76]. An American College of Obstetricians and Gynecologists report shows that at least 25 times as many women with an unmet wish for a peripartum TO have an unintended repeat pregnancy within a year than women over 30 years of age who obtained PC develop such serious regrets that they later elect to have a reversal [107]. Facilitating the desire for a TO and paying for the occasional IVF is likely to cause the greatest happiness to the greatest number of women, plus it will be often cost-effective.

The best approach for obstetricians and midwives who fear that women/couples often have too little time (e.g. CS of a 36-year-old Para 2 for a failed induction of a post-dates pregnancy, or for mild hypertension; or an unexpected large breech presentation with beginning contractions at 39 weeks) to decide about a concurrent TO, is to make early “what if you need a CS” antenatal counselling a routine.

Young age, substance misuse, medical indications for TO, coercion and unstable relationships are associated with an increased incidence of TO regret. Regret is however frequently related to unanticipated events (divorce, death of partner or child) which, in prosperous countries with routine extensive antenatal foetal ultrasound screening and even non-invasive prenatal DNA tests can’t be much better anticipated ― except in cases of prematurity when more caution is advised ― a few months post-delivery than during a CS with a paediatrician in attendance to quickly check the new-born [114].

Early counselling is frequently impossible to achieve in less-resourced circumstances where patients tend to arrive unbooked, often via a clinic and in labour, in hospitals capable of performing CSs. Nevertheless, under those circumstances the consequences (*vide infra*) of a missed TO opportunity, not in the least because there is in the meantime a scar in the uterus, are much more often dangerous [18, 38]. One wonders however ― data are impossible to obtain ―, how often counselling, that is respecting a woman’s autonomy, whether well-timed or not, occurs in the different European FIGO member countries [47, 76]. The antenatal software, or the pre-printed paper dossier, could easily be made to give a suitable prompt around, say, 34 weeks.

Troublingly, if the patient was indeed presented with the PC option or succeeded in taking the initiative, quite some hospitals and/or obstetricians and/or anaesthetists insist on a fee for a concurrent TO, as if two separate operations are needed. Poor multiparae, including immigrants [74, 75], for whom LARCs are often also too expensive, or too scary, become the victims of these questionable practices ― their obstetricians and GPs will often not hear about their subsequent visits to abortion clinics or even the backstreets, especially if it involves an immigrant later returned to a country in disarray and perhaps to her 4 children there. [76, 115]. To ensure that the decision to offer and, if accepted, to perform a TO during a CS is entirely based on beneficence, a TO during a CS should perhaps not involve extra payment to the obstetrician at all.

There is good evidence that clips for peripartum TOs ― unfortunately irresistible to gadget aficionados ― use of which makes extra payment by patient or insurance unavoidable are certainly not better than sutures while they are much more costly [7, 48, 116]. Their peripartum use is completely irrational, even more so if BTS is going to be the norm.

Although there is no proof of higher TO regret rates if CS/TO could only be discussed not long before delivery, there is evidence that this late counselling results in an increased number of regretted rejections of the TO option, and therefore unintended pregnancies [76, 96]. It would be enlightening and ethically responsible, if readers could please systematically follow up their patients with two or more children who last delivered by CS and ask them about their experiences and opinions: questionnaires to copy available online [76].

As mentioned, higher BMIs [30] and older age are associated with increased CS rates [27, 28]. Failure to inform, in time, in particular such women about their CS risk and about the inherent TO option, certainly clashes with modern medical ethics [106]. These are also the very women for whom other contraceptive options like depot-medroxyprogesterone-acetate (DMPA), combined hormonal methods, copper intra uterine device (CU-IUD) in case of sizeable fibroids (fibroids are more prevalent in African and older women and are also when large associated with higher likelihood of venous thromboembolism) tend to be (relatively) contraindicated [31].

Of course, women known to have a BRCA1 or BRCA2 gene mutation and pregnant of what they hope to be their last child, are also very much entitled to BTS counselling.

More and more women suffer from ― often age-related ― subfertility and undergo IVF treatment. These women have, when the IVF is successful, high CS rates [29]. Although counterintuitive, they are ideal candidates for CS/BTS counselling. In case they want another child, then, preferably, well-planned and quite soon ― not first struggling for months while ovaries age further, and the gametes of both partners are somewhat more likely to become damaged in vivo ― so often again via IVF. Frequently, there will still be some frozen blastocysts, or perhaps oocytes. In addition, with IVF there is an increased risk of an ectopic pregnancy, but very seldom ― an interstitial ectopic is still possible ― if there was a BTS during a previous CS [7, 117]. Couples who are very grateful that IVF/ICSI made it possible to have the family they so eagerly wanted tend to be extra disturbed when facing an unintended, completely unexpected (contraception is often thought to be unnecessary), pregnancy when she is 38–49 years old. Such women are regularly seen in abortion clinics but there is seldom feedback to the IVF doctors and to those who saw her antenatally and performed the CS [115].

Many women who are around 35 years or older and who had a CS earlier are in a similar position because they are not very likely to become pregnant again they are less prepared to undergo the disadvantages of reversible contraception or jump through the many hoops *en route* to an interval TO. For example, in Spain an estimated 840,000 (32%) women aged 40 to 50 years are at risk of an unwanted pregnancy while 52% of women in that age group use contraception: mostly (male or female) PC [118]. In the US, the data are similar. In the Netherlands 16% of the at risk in this age group is unprotected, more than three times the proportion of at risk teenagers (5%) [61].

## Less-resourced circumstances

Very disturbingly, in less-resourced regions with a large unmet need for contraception, around 225 million women are involved [80], there is a move away from FPC to other modern methods with more unintended pregnancies as result [119]. High failure rates, frightening or irritating side effects [120] could undermine the cautious trust in the competency of scientific/modern health care, which must influence compliance in other areas, such as the advice not to stop anti-TB treatment too soon, to vaccinate or to make absolutely sure to deliver in hospital if the uterus is scarred. The complications of unintended pregnancies are extra serious in those very areas [53], not least because often the anti-TOP laws were largely left untouched after decolonialisation. The result is that the overall figures show that it is in general about as dangerous to have a TOP as to continue a pregnancy with a maternal mortality of about one in 180 in SSA [121]. This while in many other countries the continuation of a pregnancy is much more likely (a factor 10 in the US) [53] to end in a maternal death than a ― legal, safe, on average much earlier in pregnancy ― TOP does.

Stock outs, lack of infrastructure and transport (many large, informal, densely populated urban settlements included), of equipment and properly-paid, well-supervised health workers, in combination with enduring pronatalist traditions, the authority of the religious leaders and de facto rural, sometimes urban, health monopolies of catholic and specific orthodox protestant hospitals frequently undermine an effective, client-friendly, integrated approach to contraception [62, 71, 89, 91, 107, 122, 123]. Without guaranteed access to reliable contraception and/or well-supervised deliveries (in SSA about half of the births are assisted by skilled birth attendants) and/or safe abortion, a CS scar in the uterus is like a landmine which can still explode 15–20 years later [96, 124–126]. CSs can moreover result in a morbidly adherent placenta in a subsequent pregnancy with fatal maternal consequences, even in high-tech US (3% mortality [36]), let alone in the district hospitals in SSA. When women have or want more children and therefore more repeat CS these risks multiply exponentially [36]. Even routine CSs can have high mortality rates in rural/regional/central hospitals [127, 128]. In the meantime, CS rates are rising also in less-resourced circumstances [24, 26, 127].

Doctors working for relief organisations, including those from countries where very high CS rates are routine [26], are performing many CSs, and they rarely combine these with FPC. They often do not ask if patients with 3–10 children would like a TO because there are language problems, they assume that women or their partners (who, by the way, if not physically present, are frequently telephonable these days) are not interested, or they are afraid of the husbands like in South Sudan and they take it for granted that it is unethical to counsel in a hurry even when there was no earlier antenatal opportunity to have a “what if you need a CS” talk.

The very fact that relief/NGO doctors are present makes early/any antenatal counselling, future access to reliable contraception, well-supervised labour after a previous CS and professional care for patients with complications of unsafe abortions problematic in both the short and long term. Tragically, having an unwanted pregnancy and therefore more often need for a “what if you need a CS” talk, tends to reduce the chance of antenatal care utilisation, just like long clinic waiting times, poor patient-provider relationships including demeaning attitudes, (food) insecurity, stigma, (transport) costs, staff shortages, cultural beliefs and lack of husband’s permission to travel [123, 128–130].

It seems rather condescending to maintain that a woman who would remember how she felt when she detected that she was pregnant again, and who has been pregnant for many months is not in the position, even as labour has started, to decide whether she would, given the choice, like to have more pregnancies [106]. Compare that with an ethically cleared randomised study under patients who just had a ST-segment elevation myocardial infarction, who are asked to participate in a study to help detect which percutaneously inserted coronary stent is superior [131]. In both cases we have to trust that the patients’ benefit ― in the latter case future patients ― is considered paramount. Working in an environment, where LARC is largely unavailable, where the lifetime risk of maternal death can be 1 in 50 as opposed to 1 in 10,000, where the mortality related to CSs is 100–600 times, the case fatality rate of TOPs 750 times higher than in the US [53, 127] and where many do not visit the antenatal clinic, should obviously affect the decision whether to give ― even between contractions ― say a Para 4 who needs a CS, the TO option or not. Perhaps a 40% under-five malnutrition/stunting rate in the area should also be a consideration, or the woman being (obviously) HIV+.

It is also important to remember that peripartum TOs if they are consented to in SSA, mostly involve women who already have 3 or more children before delivery [16, 18, 102]. For these children it is literally of vital importance that their mothers stay alive.

Arguably, asking during labour consent for a first CS (many of which are not lifesaving for the woman and have serious immediate and future risks) seems more ethically problematic than providing the TO option if a CS has already been agreed upon. The consequences of never having children again are much easier to understand for a lay person ― even for the doctor ― than all the possible future implications of having a CS, especially when not combined with a TO. Ideally, there is a partner present or telephonable who supports her either way.

Recently, hospitals and staff in East Congo, North East Kenya, West Kenya [132] Syria, South East Turkey, Iraq, Afghanistan, Eastern Ukraine, Yemen and South Sudan were prevented to work, attacked or became “collateral damage”, making it difficult to organise well-supervised labour for those with a scar in the uterus, let alone provide contraceptive services. Yet, Libya used to have a CS rate of 26.8% in 2012,Turkey 48%, Syria 19.2%, Ukraine 15.8%, Iraq 14.5%, Afghanistan 4.0%, South Sudan 3.2% and Mali 2.9%, according to estimates [26]. Combining the annual number of deliveries (825,000 (population 25 million, crude birth rate 3.3%)) and the past CS rate (4.8%), it is likely that there must be at a minimum 150,000 fertile women in Yemen with a CS scar in the uterus [133]. Before the present war 57% of the deliveries was not attended by a doctor or midwife and 38% of women who delivered did not visit the ANC even once [133]. Of the women married or living in union aged 15–49 years, 19.2% used modern contraception (global average 56.1%) of which only a third was independent ― 2.3% FPC, 4% IUD ― of (now largely defunct) supply networks for injections, pills and condoms [1]. Of the married women in Yemen, 41% does not want any more children. The total fertility rate (TFR) is 4.4 children per woman and the desired TFR 3.1 [133]. At the moment of writing, food, water, electricity, transport and fuel are very difficult to obtain, and moreover, in 2013, before the aerial attacks started, already 41% of under-fives was stunted and 44% underweight [95, 133].

During the Ebola epidemic in West Africa, routine hospital work, including antenatal and delivery care, provision of contraceptives, and anti-HIV therapy more or less came to a standstill for many months in the relevant areas. Also, it is likely that funds earmarked for HIV/AIDS assistance in SSA have negatively affected the capacity to relieve the unmet need for modern contraceptive methods: there is a serious shortfall of resources for family planning [11, 50]. Moreover, siphoning off staff into the vertical organised HIV programmes in SSA seriously interferes with basic health care, including family planning and assisting with deliveries [14, 134].

Therefore, there are many places where it borders on malpractice to perform a CS for a to-be-on-the-safe-side indication ― e.g. a routine elective CS for breech presentation or a failed misoprostol induction for a somewhat elevated blood pressure, etc. ― if a concurrent TO is not on offer or not desired by the patient. This used to be the situation in developed countries certainly hundred years ago when CSs were quite dangerous, modern contraceptives not available and TO illegal or seen as unethical [36]. This was often still the case the first 20 years after the Second World War when there was enough medical staff and equipment, antibiotics, a good infrastructure making successful referrals possible, reliable electricity, and a low incidence of obstetrical fistulae from prolonged neglected obstructed labour. Parities were often akin to those presently seen in SSA. Therefore, CS rates were low, related to the fact that CSs were about as dangerous as they are presently in many less-resourced circumstances [104, 127, 128].

Moreover, a WHO report about CSs in SSA states: “Emergency cesareans, when performed, are often too late to reduce perinatal deaths” [135]. I remember a few Para ≥4 who told me after a CS resulting in a stillbirth or an early neonatal death that at least we should have offered a TO because then they would have had some benefit from the operation: now they feared a future pregnancy. If they had delivered the deceased foetus vaginally, often easily possible (if need be with a craniotomy), then they would have had the option of a post-vaginal delivery minilap TO. Very few doctors are prepared to perform such a TO soon after a CS.

In quite some less-resourced settings, uterine ruptures, fatal obstetric haemorrhage associated with CS deliveries and obstetric fistulae develop even when women labour in health facilities [125, 128, 136, 137]. One can imagine the danger if women with a scar in the uterus avoid hospital deliveries [138] or can’t reach a reasonably equipped/staffed medical facility in time. Therefore, arguably, as long as obstetric fistulae are not uncommon in a certain region, a CS should only be performed if there is (also) a solid maternal indication, unless the patient and perhaps the family too, is properly counselled about the future risks, or alternatively, the patient elects to have a concurrent TO. It follows that, besides protesting TO counselling performed under time pressure [101] in situations where a CS scar is quite dangerous and where earlier “what if” counselling about the options in case of a CS, was not done or not feasible [96], the ethicists should also worry about the absence of emergency counselling *vis-à-vis* the potential consequences of not performing a concurrent TO [127, 128]. There is a tendency in low-resourced areas to believe, and there is outside encouragement [26], that “Western” or perhaps Chinese CS rates are needed for optimal obstetrical care [139], while the high-tech facilities, staff, drugs, contraceptives (prevalence of modern contraception in SSA is 1/4–1/3 of the global figure [1]) and the availability of safe abortions are seldom guaranteed, to limit the dangers. One step in the right direction would be that relevant women are always provided ― preferably antenatally ― by the TO option.

It would be very good if the international community would donate enough LARC. It might, in the long run, contribute more to global misery-prevention than any other assistance [10, 11, 122, 140, 141]. Of course, as stated, this is not easily implemented because of weak medical system infrastructures [57, 96, 98, 122, 134, 142]. One often reads reports about enthusiastic acceptance of IUDs, implants, and TO under local anaesthesia during donor-funded drives with generous per diem allowances for trainees and perhaps transport refunds for the clients, but integrating these services year after year in the daily routine ― postpartum or post admission after a clandestine abortion, would often be easiest for the women, plus a walk-in reproductive health clinic ― is quite another story [143, 144]. Moreover, IUDs are sometimes expulsed or they partly descend therefore they need longer and more well-equipped medical back-up than TOs [55, 145]. This also applies to IUDs fitted during a CS or within 48 h of a vaginal delivery. If the family is complete, the associated 5–10% expulsion rate, often unrecognised by the woman and in practice and when the strings are not visible not easily excluded in clinics ― most having no ultrasound or X-ray facilities ― is well-nigh unacceptable. Implants, which can be inserted before hospital discharge after delivery or after abortion/miscarriage, need very little aftercare till the next insertion but irregular bleedings can be very bothersome/fear inducing. It could be, however, that women in SSA are more prepared to accept these inconveniences, as with injectable contraceptives, because they often personally knew women who died of a pregnancy or they nearly did this themselves, and they often have no proper alternatives [140, 146].

In some less-resourced circumstances the risk of regret following PC is increased, for example in parts of India. There childbearing still starts young while desired family size has decreased substantially. Women may have what they consider a completed family at age 22–24 years. The chance of a regretted TO, because of loss of the partner or a child, is quite high in such settings, and there is seldom access to tubal reconstruction/IVF [147]. Access to LARC ― contraceptive injections are too controversial in India but popular in Thailand, Indonesia, Bangladesh and Sri Lanka [1] ― to postpone TO for 10 years or so, or, when satisfied, for continuation, would help. Alternatively, at some stage, it would probably also make economic sense for India ― renowned for “fertility tourism” ―, and also for example for Indonesia, with a 12% and fast increasing CS rate [26, 148], to have a low threshold for both TO counselling and gratis IVF provision if a partner or child has died ― as has happened in China after an earthquake killed many children [76]. Conversely, the few TOs currently performed in large areas of SSA often involve older women who regularly have 1–3 children more than originally desired [16, 18, 96, 102, 141] and who, moreover, are likely to have, orphans in the extended family who need them also [149].

In SSA, FPC is not often routinely antenatally discussed unless there is a strong medical indication like in anticipation of the 3rd-5th CS. For example, a paper from Ghana about pregnancies in HIV-infected women states: “This may call for a new approach in which healthcare providers initiate such discussions (i.e. about patients’ reproductive intentions) with women living with HIV” [150]. This suggests that women who are not known to be HIV positive will for the time being lack this very basic service.

Medical teachers and role models, mostly city-based, tend to promote the high-tech optical instrument TO approach, giving doctors in training the false impression that using basic technologies for peripartum and interval TO is lacklustre and obsolete [48]. Those young doctors could later, e.g. as district doctor in Africa or Asia, be handicapped as PC-providers because of lack of fancy instruments.

Very fortunately, otherwise some regions would have very bleak futures, and contrary to conventional wisdom, voluntary contraception will not become widespread only when standards of living and education have improved. After initial acceptance of family planning by opinion leaders, the availability of modern contraception itself, including dedicated pro-active services, drives the decrease in pregnancy rates [11, 151].

A form of government or donor incentive for staff performing vasectomies, post vaginal delivery and interval TOs ― probably not a salpingectomy during CS because that requires very little effort, and the extra payment might just sometimes turn out to be too tempting ― is advised [96, 152]. Private gynaecologists all over the world earn often hundreds of dollars with these procedures with nobody raising ethical concerns about inducements [39]. This while many would frown upon an African or Asian government doctor receiving say 25 dollars extra. As if the latter would be more inclined to compromise his/her patients’ interests [18, 153]. Many of these government doctors are allowed some additional private practice because the state is unable to provide an ― arguably ― reasonable salary. The $25 would then recompense lost income. Even very well-to-do GPs in many rich countries receive government incentives for influenza vaccinations, and for diabetes and cervix screening, while it is just an easy part of their job. One could also argue that private obstetricians in some countries have an incentive to discourage CS/TO, even if only 50% of their patients return for a hysteroscopic/laparoscopic TO later.

## HIV/AIDS

In 2009, 370,000 children became newly infected with HIV. These tragedies often started as unintended pregnancies, while the woman knew, suspected or could not exclude that she was HIV positive [71, 154–156]. By 2013, vertical HIV transmissions had declined to around 200,000, not really because there were fewer unintended pregnancies of HIV positive women but because of better access to screening and anti-HIV therapy of pregnant and breast feeding women [156]. Since the advice is to start treating HIV+ children (and recently also adults) immediately after diagnosis, the vertical HIV infected ask ― hopefully for at least eighty years per child ― a gigantic financial and manpower commitment (billions of dollars) that is unlikely to be sustainable [134, 157]. According to UNICEF, in 2014, there were 2.6 million HIV+ under-15 children globally, most of whom were vertically infected and only one in three was on treatment. AIDS is the number one cause of death in Africa among adolescents (10–19 years). Adolescent AIDS deaths have tripled since 2000. Moreover, presently 13.3 million children (0–17 years) have lost one or both parents to AIDS [158].

The above suggests yet again, that far more misery is caused by not giving potentially interested women the option of a TO if an opportunity arises, than by providing the TO option, even, if forced by circumstances, in a hurry. In many cases, any health worker who has seen the woman antenatally, or even earlier at the HIV clinic, and did not discuss and record her reproductive intentions and did not assist her to attain those goals, behaved unprofessionally: not, it is suggested, the doctor who provided, albeit between contractions, the TO option in suitable cases.

There is now a fast-growing group of HIV+ women who have a completed family, are older than 30 years and would benefit from PC. There are communities in SSA where more than 50% of pregnant women of 30 years and older are HIV positive while at the same time childbearing starts very young [159]. Although proper HIV therapy will suppress the virus so well that vertical HIV transmission becomes unlikely, stopping medication because of supply or motivation problems, an Ebola outbreak or donor fatigue, might soon result in a serious vertical transmission risk. Therefore contraception should be very reliable and preferably resistant to public disturbance and economic collapse. In SSA, combined oral contraception has very high typical failure rates [96, 146]. Three-monthly DMPA injections are however quite popular (nearly nine million users, prevalence 6.8%; the most used modern method in SSA [1]), though there is still much unmet demand [10, 146] and in practice ― with a typical first year failure rate of 6% in the US [34] where the medication would be very seldom out of stock ― often the most reliable realistic reversible option for mothers with a completed family. Except, 6% is of course too much if the consequences are potentially so very dramatic, on top of that there is a large discontinuation rate because of side effects. A study from Malawi of HIV+ women on treatment showed that after 48 months 2/3 of injection users were still using the method, the same applied to CU-IUDs [130]. A South African study of both copper IUDs and progestin contraceptive injections involving women who requested long-term protection, showed failure and discontinuation rates similar to the above [160]. There is some evidence that DMPA increases the risk of horizontal HIV transmission and acquisition to a degree [160–162]. Thorough risk-benefit calculations, however, do not come out in favour of abolishing DMPA under most circumstances [146]. The injections prevent many maternal deaths and vertical HIV infections by averting unintended pregnancies [10, 96]. But these nuances might get lost when tabloids and/or persons with different agendas become involved, which has happened before in relation to this method [143], see also Wikipedia. Women with a completed family who have been offered and have accepted an opportunistic TO might consider themselves exceptionally lucky in future if DMPA is unobtainable because of physicians’ and nurses’ strikes, because USAID lost funding or because of civil war with perhaps many cases of rape [163, 164].

It might also be a good idea to offer family planning clients HIV tests ― if it does not stigmatise and hence deter visitors ― in order to perhaps adjust the method advised in accordance with the HIV test result and inform clients about the PC-option in case an opportunity present itself in future.

## Men

Of course, what’s good for the goose is good for the gander. Men in some West European countries, such as France, have very low vasectomy rates compared to those in comparable countries (US, Canada (including the Québécois), United Kingdom, Australia, New Zealand, South Korea, Switzerland, Czech Republic, Spain, Netherlands, Denmark) [1]. These are historical, legal and cultural coincidences, which need, it is suggested, evidence-based reconsideration. Other countries with sizeable vasectomy prevalence percentages are Bhutan (13.6), Nepal (6.3), China (4.5), Puerto Rico (5.3) and Brazil (5.1) [1]. Men can, not seldom the more educated ones do, contribute with vasectomies. It’s only fair, i.e., their partners were troubled for years as a result of their reproduction-friendly evolutionary design, by bothersome periods, the final responsibility ― in practice ― for contraception, including often the side effects and costs, perhaps TOPs, miscarriages, nausea, painful deliveries, breast afflictions, striae, candida infections, cystitides, cervical screenings and other inconveniences. Opportunistic vasectomies are sometimes an option in combination with inguinal hernia and hydrocele operations. Moreover, 10 million voluntary male circumcisions were performed in the years before 2016 (the aim was 20.9 million) to reduce HIV transmission in high-risk countries [165]. Perhaps some men ― but most would be too young ― would have been interested in a concurrent vasectomy.

## Conclusion

Facilitating the prevention of unintended pregnancies, which should involve inquiring about reproductive intentions, is one of the easiest and most important contributions doctors, midwives and nurses can make to individual and collective misery prevention. Without extra efforts, in which a larger role of PC appears essential, the United Nations 2030 Sustainable Development Goals [166] will not be attained. Perhaps population pressures and the associated upheavals will destabilise whole (sub) continents, in turn making it much more difficult to provide reproductive health services [109, 167]. Although around 270,000 maternal deaths and an estimated 230 million births ― 1.7 times the current number of livebirths ― are already averted annually by present global contraceptive use [12] another 21 million deliveries, and 26 million TOPs [23], would probably be avoided if the unmet need for reliable contraception was satisfied [80].

Opinion leaders, including doctors in countries with high birth rates, should propagate/demonstrate the idea that quality often tops quantity of offspring because it results in more resources per child including better education and less overall poverty. There are no medical reasons, but PC is exceedingly rare in some countries, while in others, PC (male or female) protects more than 40% of the women who are married or living in union. Globally, the average is estimated to be 21.3%. Furthermore, when comparing countries where PC is common, one notices enormous variations in the relative prevalence of male (highest in Canada, 22.0%) and female (highest in the Dominican Republic, 47.4%) PC [1]. Therefore, there must be scope, if excellent services are made available, to provide more PC services, especially in Africa, Europe and the Middle East.

Relying on PC is a very convenient state of affairs for many with a completed family. It provides peace of mind. However, the need for an operation can be quite a barrier (fear, costs, logistics, scarcity of relevant professionals), when female PC is not combinable with a delivery, especially a CS, or abortion. PC is certainly not just suitable for the poor. There are millions of well-to-do couples in places like the UK, Spain, Finland, Norway, Switzerland, North America, Latin America, East Asia, Thailand, Australia and New Zealand, quite satisfied with either male or female PC [1]. On the other hand, it is not that difficult in well-resourced circumstances with accessible TOP services for a large part of the population to attain its reproductive goals safely without PC ― although it typically involves more stress, side effects and unintended pregnancies. However, there are often disadvantaged women with a completed family in rich countries for whom it is rather difficult to have no unintended children without PC [17, 92]. Poor women in the US have an unplanned birth nearly seven times as often as higher-income women [57]. Overall, TOP rates in developed countries have declined recently but there is a shift towards married women and, for example, in England and Wales over de last 10 years TOP rates of women over 30 increased significantly [23, 168]. European women are more often married (70%) than North American women who have TOPs (41%) [23]. There are many contributing factors but it seems likely that the large difference in PC prevalence ― 5.6% and 36% respectively [1] ― is important, because many/most unwanted pregnancies in these continents are related to inconsistent or imperfect use or failure of reversible contraception [23, 53, 57].

Women who aim to have few children will not easily succeed in less-resourced countries without PC, unless they have ― often unsafe ― TOPs [13, 80, 82]. Imagine a mother of five (whose first, fourth and fifth pregnancy started as unintended conceptions) expected to travel 30 km to the nearest clinic every three months from age 32 till 50 for contraceptive injections, which may be sometimes out of stock, while transport costs reduce food and schooling available to her children. Perhaps she will develop a contra-indication like high blood pressure at age 35. This woman delivered by CS the last time but she was not given the option of a concurrent TO, and the doctor who operated her will rarely hear about her plight, just like there is often no feedback in developed countries [115].

As discussed, recent developments have made PC a more attractive option for more women, with less risk of regret and failure, mainly because employed and more-educated ― and therefore presumably less coercible ― women in many places are older when they consider their family complete, and because child mortality has decreased substantially ― but there is a risk that population growth will reverse progress and then this window of opportunity might close.

Increasing CS rates have made PC accessible to more mothers while those extra CSs simultaneously result in more women for whom it can be dangerous not to have a TO. Especially women without guaranteed access to reliable contraception, well-supervised labour and/or safe abortions run a serious risk with a scarred uterus even in referral hospitals [169]. Providing the relevant women/couples with the option of a total salpingectomy might save more money on unneeded contraceptive methods/consultations, TOPs, treatments of the complications of unsafe TOPs, and ovarian cancer therapy, than it might cost to provide IVF ― increasingly available in capitals ― to the few with serious regretted PC. Few health systems can afford to squander PC counselling opportunities.

During pregnancy, “what if you need a CS” explorations are needed about the perinatal PC option, if at all possible weeks before the expected delivery date, as FIGO advises [96, 101]. Yet denying women/couples that choice is an unethical and careless routine in many countries. The antenatal software/paper records should be adapted to prompt well-timed counselling and information leaflets, and informational posters ought to be provided in the antenatal clinics. The media also have an educational role to play.

## Abbreviations

BTS: Bilateral total salpingectomy
CS: Caesarean section
CU-IUD: Copper intra uterine device
DMPA: Depot-medroxyprogesterone-acetate
EU: European Union
FIGO: International Federation of Gynecology and Obstetrics
FPC: Female permanent contraception
LARC: Long-acting reversible contraceptives
LDCs: Least Developed Countries
PC: Permanent contraception
SSA: Sub-Saharan Africa
TFR: Total fertility rate
TO: Tubal occlusion
TOP: Termination of pregnancy
US: United States
